# Reply to the letter ‘Beyond overdiagnosis: reframing autism prevalence’

**DOI:** 10.1016/j.jped.2025.101469

**Published:** 2025-11-08

**Authors:** Kamila Castro, Rudimar Riesgo, Carlos Gadia

**Affiliations:** a Child Neurology Unit, Pediatrics Service, Hospital de Clínicas de Porto Alegre (HCPA), Porto Alegre, RS, Brazil; b Translational Research Group in Autism Spectrum Disorders (GETTEA), Universidade Federal Do Rio Grande Do Sul (UFRGS), Porto Alegre, RS, Brazil; c Nicklaus Children’s Hospital, Miami, USA

Dear Editor,

We would like to begin by sincerely thanking Hauck and Oliveira for their thoughtful comments and generous acknowledgment of the contribution of our review [1]. We share their view that discussions about autism must go beyond epidemiological concerns, encompassing conceptual, clinical, and sociocultural dimensions in order to advance scientific understanding and clinical practice in diverse contexts. Our central aim was to provide a multifactorial and critical synthesis to help qualify this important debate.

We also appreciate the valuable emphasis placed on the categorical versus dimensional debate, which we agree is both legitimate and necessary for the field to move forward. In our review, we chose to focus primarily on the factors that currently affect clinical practice and epidemiological estimates, both of which remain grounded in classificatory systems. While dimensional models represent a promising direction, they are not yet standardized or readily applicable to large-scale public health planning. Similarly, we recognize that diagnostic boundaries do influence prevalence rates. Our intention, however, was to emphasize that even within the current categorical framework, multiple methodological, clinical, and sociocultural factors already distort reported estimates. In this context, raising the issue of possible overdiagnosis does not mean denying heterogeneity or the potential value of revising nosological systems, but rather calling attention to the risks of misapplied diagnostic practices. As we noted in our review, the challenge is not to diagnose more or less, but to diagnose better.

We also welcome the expansion of the debate toward alternative perspectives that emphasize the spectrum, heterogeneity, and overlap with comorbidities. Considering autism in terms of diverse profiles resonates with our own analysis and opens important avenues for integrating patient, family, and professional perspectives. Nevertheless, for practical purposes — epidemiology, health policies, and diagnostic protocols — we must still operate within formal classificatory systems. This does not mean, however, that these systems are static; they undergo continuous updates and improvements over time, reflecting scientific advances and the evolving understanding of clinical practice. Our article seeks to provide an analysis that is useful for this present reality, while at the same time acknowledging the complexity of the phenomenon and the value of exploring complementary frameworks.

Recent findings reinforce this perspective. Litman et al. (2025) analyzed more than 5000 autistic children and demonstrated that cases clinically grouped under the ASD label actually decompose into four phenotypic classes, each with distinct genetic programs and developmental trajectories. Such advances hold promise for the development of more targeted interventions and for the identification of robust biomarkers that can refine both research and clinical practice [2].

In conclusion, we reaffirm that our intention was to contribute a critical, evidence-based perspective that can inform clinical and policy decisions in the current landscape. We are grateful for the opportunity to engage in this dialogue, and we are confident that such exchanges will advance the field in constructive and meaningful ways.

Sincerely.

## Funding source

N/A.

## Data availability

The data that support the findings of this study are available from the corresponding author.

## Conflicts of interest

The authors declare no conflicts of interest.
